# Sequential pulmonary functions in survivors of leptospirosis pulmonary haemorrhage syndrome: a prospective cohort study

**DOI:** 10.1186/s41182-024-00665-6

**Published:** 2024-12-19

**Authors:** Dilshan Priyankara, Pramith Ruwanpathirana, Roshan Rambukwella, Nilanka Perera

**Affiliations:** 1 Medical Intensive Care Unit, National Hospital of Sri Lanka, Colombo, Sri Lanka; 2 Professorial Unit in Medicine, National Hospital of Sri Lanka, Colombo, Sri Lanka; 3 Provincial Department of Health Services, Kandy, Central Province Sri Lanka; 4 Department of Medicine, Faculty of Medical Sciences, University of Sri Jayewardenepura, Nugegoda, Sri Lanka

**Keywords:** Leptospirosis, Lung function, Pulmonary haemorrhage, FVC, FEV1

## Abstract

**Background:**

Leptospirosis, a spirochaete infection, can lead to Leptospirosis Pulmonary Haemorrhage Syndrome (LPHS), which requires intensive care admission and has a high mortality. Although data on short-term outcomes are available, the long-term respiratory sequelae of LPHS survivors are not known. We aimed to identify the post-discharge pulmonary functions and functional limitations in survivors of LPHS.

**Methods:**

We conducted a prospective cohort study from January to December 2022 at the Medical Intensive Care Unit (ICU) of the National Hospital of Sri Lanka to assess the sequential changes in the spirometry parameters in patients who survived LPHS. The Forced Vital Capacity (FVC) and Forced Expiratory Volume in 1 s (FEV1) were measured on the day of discharge from the ICU (D0), 7th day after discharge (D7) and 28th day after discharge (D28). The predicted lung volume was calculated using the gender, age and height as per standard protocol. Physical and functional role limitations were assessed on D28 using the modified Medical Outcomes Study Questionnaire Short Form 36 Health Survey (SF-36).

**Results:**

Twenty-one patients with a mean age of 44 years (SD 16.07) were enrolled for the study. The majority were male patients (*n* = 19, 90.5%). Leptospirosis was serologically confirmed in all individuals. Seventeen (81%) patients had reduced FEV1 and FVC on D0, indicating a restrictive lung abnormality. FVC and FEV1 improved during the first 7 days (*p* < 0.01) but did not change significantly afterwards. Only seven individuals (33.3%) achieved a normal FVC (exceeding 80% of the predicted volume) at D28. However, 19 (90.5%) individuals achieved a normal FEV1 (exceeding 80% of predicted volume) by D28. In our study, administering corticosteroids during ICU stay did not impact lung recovery in FVC (*p* = 0.521) or FEV1 (*p* = 0.798). The participants did not have significant physical, functional, and role limitations at D28.

**Conclusions:**

The spirometry measurements of individuals diagnosed with LPHS significantly improved during the first 7 days. Most survivors did not have a functional impairment despite the FVC not recovering to normal by D28.

**Supplementary Information:**

The online version contains supplementary material available at 10.1186/s41182-024-00665-6.

## Introduction

Leptospirosis is a tropical spirochaetal zoonotic infection leading to multi-organ involvement [1]. It predominantly affects resource-poor populations, with a case fatality rate of 6.85% [1, 2]. Among the myriad of pulmonary manifestations, leptospirosis pulmonary haemorrhage syndrome (LPHS) contributes significantly to mortality [2, 3]. The incidence of LPHS ranges from 10 to 70% [4, 5] within the first week of illness [4]. LPHS is an early indicator of severe disease [6, 7]. Most patients with LPHS require intensive care admission and ventilatory support, and LPHS carries a mortality rate of 30–60% [8, 9]. The pathophysiology of LPHS is assumed to be due to pulmonary capillaritis [8].

Studies reveal that patients with pulmonary haemorrhage due to underlying vasculitis develop persistent lung function abnormalities, commonly a restrictive lung pathology and an obstructive lung pathology occurring occasionally [9]. Interstitial fibrosis following the resolution of haemorrhage is thought to cause restrictive pulmonary pathologies. Bronchiolitis or emphysema is believed to occur due to damaged small airways and lung parenchyma from reactive oxygen species and proteolytic enzymes [10].

Although short-term outcomes of LPHS are poor, the long-term sequelae of survivors are yet to be studied. The impact of LPHS on lung function after recovery is not known, and it is important to identify whether survivors are affected with long-term respiratory morbidity. We studied the sequential changes in lung functions of survivors of LPHS over 1 month and correlated them with physical disability. This prospective cohort study was conducted on survivors of LPHS in an intensive care unit in Sri Lanka.

## Methods

### Study setting

This prospective cohort study was conducted in the Medical Intensive Care Unit (MICU) of the National Hospital of Sri Lanka. Leptospirosis is endemic in Sri Lanka. The MICU is a referral centre for severe leptospirosis patients requiring intensive care treatment in Western Sri Lanka.

### Study period

The study was conducted from 01/01/2022 to 31/12/2022. A period of 6 months where the MICU was dedicated for COVID-19 patients was not included.

### Study population

All survivors of LPHS with a serological confirmation of leptospirosis were recruited for the study.

### Method of diagnosis

Leptospirosis was diagnosed with either a positive polymerase chain reaction (PCR) for *Leptospira interrogans* DNA in the serum or a microscopic agglutination test (isolated titre of ≥ 1:320 or fourfold rise/seroconversion from acute to convalescence) for anti-leptospira antibodies in the serum. Pulmonary haemorrhage was diagnosed if a patient developed haemoptysis (or blood-stained secretions from the endotracheal tube), hypoxia (SpO_2_ < 94%) with bilateral fluffy opacities in the lung parenchyma in a chest radiograph, or evidence of pulmonary haemorrhage in high-resolution computed tomography and a reduction of haemoglobin more than one g/dL within 48 h. Patients who had pre-existing chronic pulmonary diseases were excluded from the study.

### Study methods

The demographic details, anthropometry (height and weight), clinical features, laboratory parameters, and treatment received were extracted from the clinical notes and entered into an electronic database. Participants were prospectively followed up for 28 days after discharge.

All patients were treated with intravenous antibiotics, plasmapheresis and intra-nasal desmopressin as per local protocol. Some patients received intravenous methylprednisolone (dose ranging from 0.5 g to 1 g per day for 1–3 days) as per physician discretion. None of the patients were discharged with steroids, non-steroid immunosuppressants or antifibrotics.

### Pulmonary function assessment

A trained technician measured the Forced Vital Capacity (FVC) and Forced Expiratory Volume in the 1st second (FEV1) on the day of discharge from MICU (D0), 7 days after discharge (D7) and 28 days after discharge (D28) using a ‘SpiroScout’ ultrasound desktop spirometer. Three repeated measurements were taken at each instance, and the highest value for each parameter was used for the analysis. FEV_1_/FVC ratio was calculated for each time point. The same spirometer was used to record all measurements. Study participants who died within the first 28 days after discharge were excluded from the analysis.

The predicted lung volumes for each individual were calculated using height and age per standard protocol [10, 11]. The measured FVC and FEV1 values were expressed as a percentage of the predicted value for each individual (FVC% and FEV1%).

### Assessment of the functional status

We used a modified version of the validated modified Medical Outcomes Study Questionnaire Short Form 36 Health Survey (SF-36) [10, 11] to assess the LPHS-related quality of life on D28. We used the two domains (questions 4 and 5) most relevant to this study, assessing physical function and role limitation due to physical health. We scored each patient per ‘The SF-36 Health Survey Manual and Interpretation Guide [10]. A higher score indicated a better functional status.

### Statistical analysis

Statistical analysis was performed using IBM SPSS Statistics for Windows, version 20.0.2.0. The continuous variables were described using mean (standard deviation) or median (interquartile range) based on the data distribution. Categorical variables were described as percentages and compared using the Chi-square test.

The sequential changes of the spirometry parameters were analysed using repeated measures one-way ANOVA. Variables affecting the change in lung function were assessed using a mixed model repeated measures ANOVA. A Bonferroni adjustment was done, where multiple comparisons were conducted.

### Ethics statement and consent

Ethical approval was obtained from the ethics review committee of the National Hospital of Sri Lanka. Patients were recruited to the study on discharge from the MICU (D0) after obtaining informed written consent.

## Results

Twenty-one patients were recruited with a mean age of 44 (SD = 16.07) years. All patients had a serologically confirmed diagnosis of leptospirosis, and LPHS was diagnosed based on the criteria described above.

The majority were males (male-to-female ratio 9.5). The median weight was 55 kg (IQR 43.5–68), and the mean height was 1.62 m (SD = 0.08). Table 1 gives the characteristics of the study population, clinical parameters, and treatment strategies.

**Table 1:** Summary of patient characteristics

Characteristic	Value *N* = 21
Co-morbidities, *n* = 8 (38.0%)	
• Diabetes mellitus • Hypertension • Psychiatric illness	6 (28.6%) 5 (23.8%) 1 (4.8%)
Disease characteristics on admission to MICU	
Systolic blood pressure (mmHg)	112.9 (27.3)*
Diastolic blood pressure (mmHg)	67 (60–70)*
Heart rate (beats/min)	98 (79.5–124)*
White blood cells (× 10^3^/µL)	12 (7.9–15.8)*
Haemoglobin (g/dL)	9.79 (1.68)
Platelets (× 10^3^/µL)	35 (20.5–56.5)*
Sodium (mmol/L)	137 (132.05–140)*
Potassium (mmol/L)	3.87 (0.67)
CRP (mg/dL)	214.38 (138.24)
Serum creatinine (mg/dL)	3.39 (1.94)
AST (U/L)	98 (52.5–128)*
ALT (U/ L)	55 (42.5–96)*
APTT (s)	36.56 (8.80)
INR	1.12 (1.0–1.39)*
Lactate (mmol/L)	2.0 (1.15–3.05)*
Base excess (mmol/L)	− 5.32 (6.06)
PaO_2_/FiO_2_	201.6 (73.4)
Treatment strategies, *n* (%)	
Intravenous methylprednisolone,	6 (28.6)
Duration of ICU stay (days)	4 (3–6)
Duration of hospital stay (days)	6 (3.5–7.5)*

Values are given as mean (SD) or *n* (%). *Indicate values expressed as median (IQR)

*AST* aspartate transaminase, *ALT* alanine transaminase, *INR* international normalized ratio, *APTT* activated partial thromboplastin test, *PaO*_*2*_ partial pressure of arterial oxygen, *FiO*_*2*_ fraction of inspired oxygen

Eight (39.1%) patients were mechanically ventilated for a median duration of 4.5 days (3–6). All patients received oxygen therapy via face mask, high-flow nasal oxygen, continuous positive-pressure ventilation and non-invasive ventilation at different stages of the illness. The median duration of oxygen therapy was 4.5 days (3–7.5). None received extracorporeal membrane oxygenation (ECMO).

**Fig. 1:**
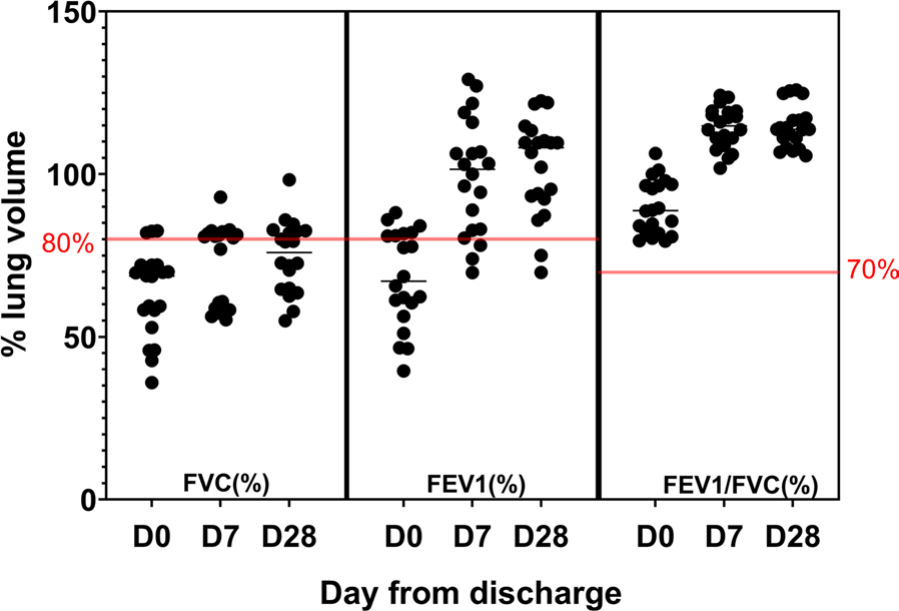
Sequential changes in spirometry parameters in the study population. Pulmonary function was assessed in survivors of leptospirosis pulmonary haemorrhage syndrome on discharge from the ICU (D0), on the 7th day (D7) and 28th day from discharge (D28). The figure demonstrates forced vital capacity (FVC), forced expiratory volume in the 1st second (FEV1), and FEV1/FVC ratio in each patient as a percentage of the predicted value. The horizontal red line indicates 80% and 70% are the lower cut off of the normal range for FVC, FEV1 and FEV1/FVC ratio, respectively

The median spirometry parameters on D0, D7 and D28 are given in Table 2. Seventeen (81%) patients had a restrictive lung abnormality on D0. Lung functions were normal in three (14.3%) patients. We excluded one patient from the analysis as the lung volumes significantly deviated from the other participants at all three timepoints. The sequential spirometry values expressed as a percentage of the predicted value for their age, gender and height are given in Fig. 1.

**Table 2:** Sequential changes of mean/median spirometry parameters

Spirometry parameter	D0		D7		D28	
	**Absolute volume/ ratio**	**As a percentage of predicted volume**	**Absolute volume/ ratio**	**As a percentage of predicted volume**	**Absolute volume / ratio**	**As a percentage of predicted volume**
**FVC (L)**	2.4 (1.5–2.6)	68.8 (55.5–72.1)	2.6 (1.9–2.9)	80.4 (58.8–82.0)	2.5 (2.1–2.8)	80.4 (64.1–82.5)
**FEV1 (L)**	2.1 (1.3–2.4)	65.62 (56.1–81.4)	2.7 (0.6)*	102.9 (82–117)	3.2 (2.2–3.3)	109.6 (92–114)
**FEV1/FVC (%)**	86.9 (81.2–96.4)		85.7 (82.8–90)		87.8 (80.8–91.2)

A repeated measures one-way ANOVA showed that FEV1 (*p* < 0.001) and FVC (*p* = 0.008) improved significantly over 1 month. A significant improvement in the FEV1 and FVC was observed from D0 to D7 in a post hoc analysis. Increments of the FVC (*p* = 0.853) and FEV1 (*p* = 0.884) from D7 to D28 were not statistically significant (*p* > 0.05).

Although FEV_1_ and FVC increased over time, median FVC did not improve beyond 80% of the predicted value at D7 or D28. Median FEV_1_ reached the predicted value by D7 (Fig. 1). Only 33.3% (*n* = 7) of the patients reached FVC values above 80% of the predicted at D28, while 90.5% (*n* = 19) reached FEV1 above 80% of the predicted value for each individual.

**Fig. 2:**
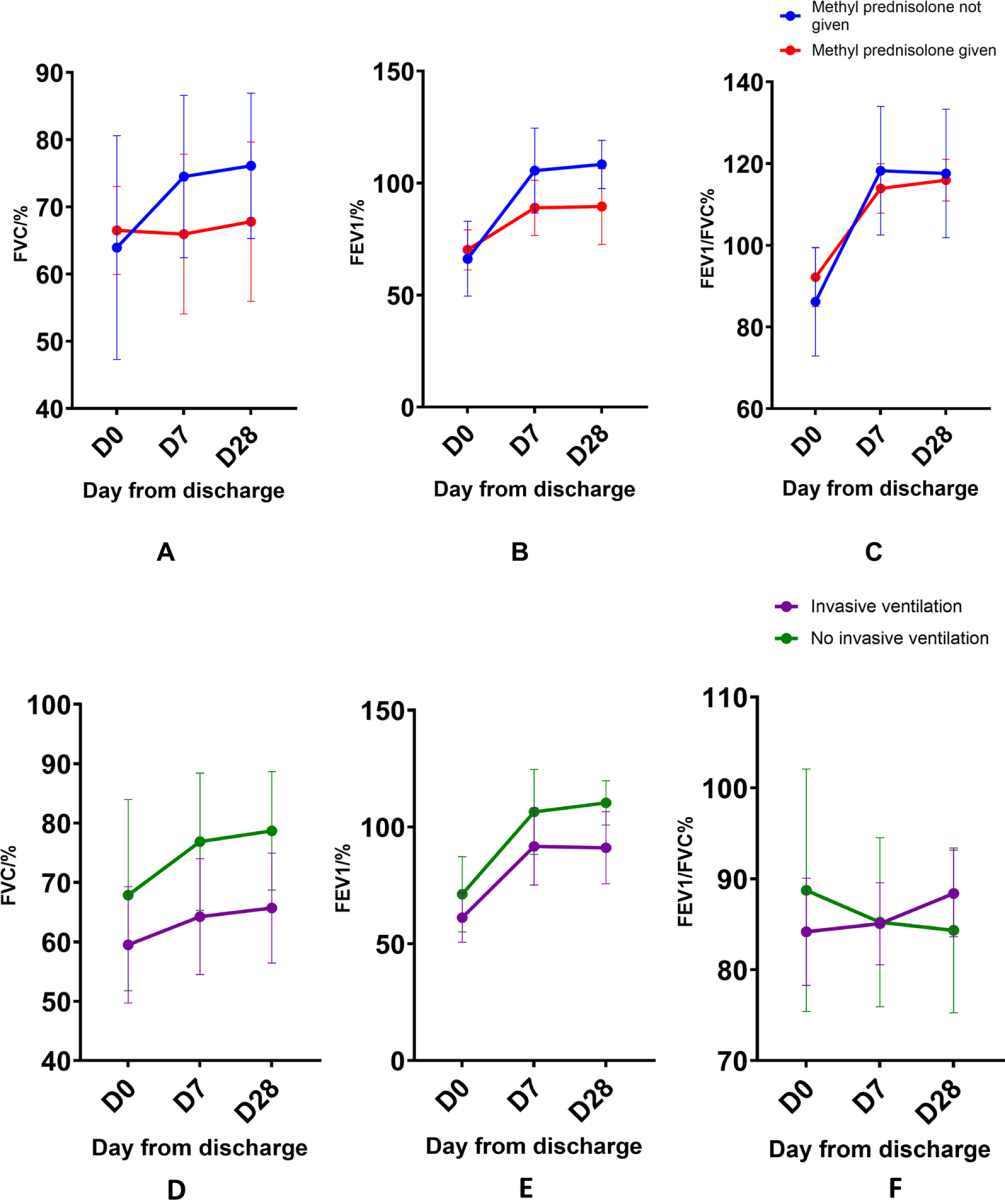
Sequential changes of pulmonary function in patients grouped according to treatment with steroids (**A–C**) and invasive ventilation (**D, E**). (**A** & **D**) FVC, (**B** & **E**) FEV1 and (**C** & **F**) FEV1/FVC ratio are given as a percentage of the predicted value for age, sex and height. D0; day of discharge from the ICU; D7; 7th day after discharge; D28; 28th day after discharge. Methyl-prednisolone was given intravenously with a dose ranging from 0.5 g to 1g per day for 1–3 days

Gender [FVC (*p* = 0.465), FEV1 (*p* = 0.555)], treatment with methyl-prednisolone [FVC (*p* = 0.521), FEV1 (*p* = 0.798)], mechanical ventilation [FVC (*p* = 0.236), FEV1 (*p* = 0.203)], and development of hospital-acquired infection during ICU stay [FVC (*p* = 0.990), FEV1 (*p* = 0.972)] was not associated with the post-discharge changes in lung volumes, in our study. The sequential changes in lung functions of patients treated with intravenous methylprednisolone compared to patients who did not receive methylprednisolone are given in Fig. 2.

Patients with longer hospital or ICU stays and longer durations of mechanical ventilation exhibited lower spirometry values at day 28 (expressed as a percentage of predicted values).

A significant negative correlation was observed between FVC on day 28 and hospital stay [Spearman’s rho (r_s_) = − 0.554, *p* = 0.009], ICU stay (r_s_ = − 0.565, *p* = 0.008) and duration of mechanical ventilation (r_s_ = − 0.477, *p* = 0.029). Similarly, FEV1 on day 28 was negatively correlated with hospital stay (r_s_ = − 0.457, *p* = 0.037), ICU stay (r_s_ = − 0.522, *p* = 0.015) and duration of mechanical ventilation (r_s_ = − 0.565, *p* = 0.008). A graphical illustration is given in supplementary material 1. There was no significant correlation between the duration of oxygen therapy and spirometry values at day 28."

Table 3 gives the functional status and role limitations of study participants at D28 identified by the SF-36 tool.

**Table 3:** Activity and role limitation at D28 (modified SF-36 questionnaire)

Activity limitation (domain 4)	Limited a lot *n* (%)	Limited a little *n* (%)	Not limited at all *n* (%)
Vigorous activities, such as running, lifting heavy objects, participating in strenuous sports	0	14 (66.7%)	7 (33.3%)
Moderate activities, such as moving a table, pushing a vacuum cleaner, bowling, or playing golf	0	10 (47.6%)	11 (52.4%)
Lifting or carrying groceries	0	10 (47.6%)	11 (52.4%)
Climbing several flights of stairs	0	3 (14.3%)	18 (85.7%)
Climbing one flight of stairs	0	0	21 (100%)
Bending, kneeling, or stooping	0	0	21 (100%)
Walking more than a mile	0	0	21 (100%)
Walking several blocks	0	0	21 (100%)
Walking one block	0	0	21 (100%)

Limitation of functional role (domain 5)	Yes *n* (%)	No *n* (%)
Cut down the amount of time you spent on work or other activities	14 (66.7%)	7 (33.3%)
Accomplished less than you would like	9 (42.9%)	12 (57.1%)
Were limited in the kind of work or other activities	7 (33.3%)	14 (66.7%)
Had difficulty performing the work or other activities (for example, it took extra effort)	6 (28.6%)	15 (71.4%)

The median physical activity and functional role scores at D28 were 94.4% (83.3–100) and 75.0% (0–100%), respectively. There was no statistically significant correlation between the lung volumes and the activity and functional role scores.

## Discussion

LPHS carries high mortality [12], but the long-term sequelae of survivors are not known. It is imperative to see whether pulmonary haemorrhage results in persistent lung abnormality and a limitation in functionality. This is the first prospective cohort study that describes the pulmonary functions and functional limitations in survivors of LPHS. Patients had a restrictive lung defect at the time of discharge from the ICU. The lung volumes improved significantly over the 1st 28 days in survivors of LPHS, with a marked improvement observed over the first 7 days. The improvement was best in FEV1. The majority did not achieve a normal FVC by D28. There was no functional limitation at the end of 28 days. Multi-organ involvement in leptospirosis and duration of ICU stay did not affect the recovery of pulmonary function.

Patients who recovered from pulmonary haemorrhage had a reversible restrictive lung pathology on discharge from the ICU. This is consistent with previous reports of restrictive pulmonary function defects in vasculitis or autoimmune disease-related diffuse alveolar haemorrhage [13–15]. Recurrent pulmonary haemorrhage may result in interstitial fibrosis, which could lead to a residual restrictive pulmonary abnormality. The neutrophil infiltrate in the alveoli causes free radical-induced airway and parenchymal damage, leading to bronchiolitis or emphysema [8]. The pathophysiology of LPHS is not clearly understood. Leptospira antigens have been detected in the lung tissue in patients with LPHS [2], and the trigger of lung injury is presumed to be Leptospira antigens or toxins/mediators related to the organism [5]. Pulmonary capillary involvement is suggested by endothelial cell swelling, increased pinocytotic vesicles, giant dense bodies in the cytoplasm, and emission of pseudopods [17].

The improvement in lung functions can be explained by the subsidence of inflammation, clearance of red cell debris and improvement of respiratory muscle function. However, as the FVC did not return to normal, we do not know whether permanent lung damage exists. Whether the FVC would continue to improve, or the presence of lung fibrosis should be sought in further studies with long-term follow-up utilising lung imaging. According to our study, the long-term implications of LPHS are minimal in terms of physical functionality. A strategy to screen LPHS survivors may be useful in capturing patients with residual lung function abnormalities.

Diseases with recurrent pulmonary haemorrhages (like idiopathic pulmonary hemosiderosis) are well known to cause lung fibrosis and respiratory failure [16]. In a cohort of 10 patients with ANCA associated vasculitis or systemic lupus erythematosus who developed pulmonary haemorrhages three patients developed an obstructive respiratory pattern, and five patients developed a restrictive pattern [15]. Two patients had had multiple episodes of pulmonary haemorrhages, and the spirometry was done within 1–10 years from the diagnosis.

Data on long term pulmonary functions in patients who develop a single episode of pulmonary haemorrhage is sparce. The impact of pulmonary haemorrhage in Goodpasture’s disease (a monophasic illness) is believed to be minimal in the long term [17].

We have recruited all patients who presented to the main reference centre for LPHS within the study period to cover the Western, Northwestern and a part of the Southern province of Sri Lanka. There were no patients lost to follow-up in our study. A limitation was the absence of baseline lung function parameters in the study participants. Values were interpreted based on the predicted values. A longer follow-up beyond D28 would have helped to identify further improvements in the FVC values of participants. Respiratory muscle weakness (due to critical illness, myositis, etc.) would have contributed to the lung function abnormalities, at least at the time of discharge. We could not perform specific tests to assess the respiratory muscle power. The functional limitations observed in a minority could be multifactorial and might not reflect a direct effect of pulmonary haemorrhage.

## Conclusions

LPHS result in a restrictive pulmonary function abnormality in the majority, which resolves in the first week and stabilises 28 days after recovery. Survivors of LPHS do not have significant limitations in physical activity or role limitations after recovery.

FVC; Forced Vital Capacity, FEV1; Forced Expiratory Volume in 1 s. * Value is given as mean (SD). All other values are given as median (IQR).

## Supplementary Information

**Additional file 1:** The correlation between the spirometry parameters and the duration of hospital stay (A&D), ICU stay (B&E) and mechanical ventilation (C&F). The spirometry parameters had a negative correlation with the duration of hospital stay, ICU stay and mechanical ventilation.

## Data Availability

The datasets used in the current study are available from the corresponding author upon reasonable request.

## Abbreviations

LPHS: Leptospirosis pulmonary haemorrhage syndrome
FVC: Forced vital capacity
FEV1: Forced expiratory volume in the 1st second
DLCO: Diffusing capacity of the lungs for carbon monoxide
HRCT: High-resolution computed tomography
AST: Aspartate transaminase
ALT: Alanine transaminase
INR: International normalised ratio
APTT: Activated partial thromboplastin test
PaO_2_: Partial pressure of arterial oxygen
FiO_2_: Fraction of inspired oxygen
