# Supplementary figures and images for: Sequential pulmonary functions in survivors of leptospirosis pulmonary haemorrhage syndrome: a prospective cohort study
Source: Trop Med Health. 2024 Dec 19;52:96. doi: 10.1186/s41182-024-00665-6 (PMC11657222; doi:10.1186/s41182-024-00665-6)

## Slide 1
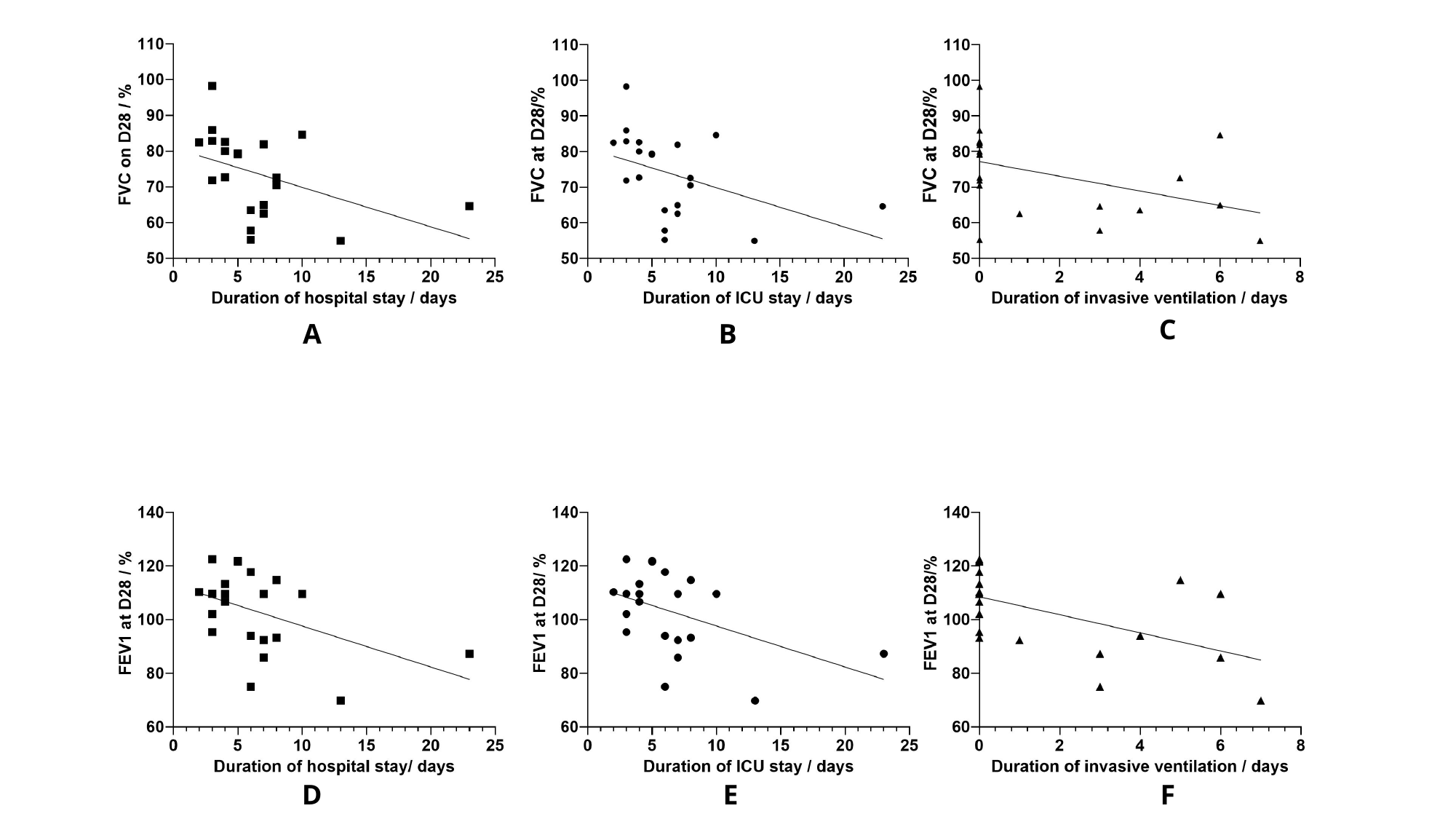

C
A
B
D
E
F

Supplement: Supplementary file 1 — Additional file 1: The correlation between the spirometry parameters and the duration of hospital stay (A&D), ICU stay (B&E) and mechanical ventilation (C&F). The spirometry parameters had a negative correlation with the duration of hospital stay, ICU stay and mechanical ventilation. [file 41182_2024_665_MOESM1_ESM.pptx]
